# Preclinical Evaluation of Puerarin as a Modulator of Metabolic and Inflammatory Parameters in a High-Fat-Diet-Fed Mice Model

**DOI:** 10.3390/molecules30193895

**Published:** 2025-09-26

**Authors:** Luisa Guadalupe Camacho-Padilla, Adelaida Sara Minia Zepeda-Morales, Javier Arrizon, Mario Eduardo Flores-Soto, José Sergio Zepeda-Nuño, Lucrecia Carrera-Quintanar, Azucena Herrera-González, Gerardo Aparicio-García, Rocío Ivette López-Roa

**Affiliations:** 1 Laboratorio de Investigación y Desarrollo Farmacéutico, Departamento de Farmacobiología, Centro Universitario de Ciencias Exactas e Ingenierías, Universidad de Guadalajara, Guadalajara 44430, Jalisco, Mexico; luisa.camacho5156@alumnos.udg.mx; 2 Laboratorio de Análisis Clínicos e Investigación Traslacional, Departamento de Farmacobiología, Centro Universitario de Ciencias Exactas e Ingenierías, Universidad de Guadalajara, Guadalajara 44430, Jalisco, Mexico; adelaida.zepeda@academicos.udg.mx; 3 Biotecnología Industrial, Centro de Investigación y Asistencia en Tecnología y Diseño del Estado de Jalisco A.C. Unidad Zapopan, Zapopan 45019, Jalisco, Mexico; jparrizon@ciatej.mx; 4 Laboratorio de Neurobiología Celular y Molecular, División de Neurociencias, Centro de Investigación Biomédica de Occidente (CIBO), Instituto Mexicano del Seguro Social, Guadalajara 44160, Jalisco, Mexico; mariosoto924@yahoo.com.mx; 5 Departamento de Microbiología y Patología, Centro de Investigación y Diagnóstico de Patología, Centro Universitario de Ciencias de la Salud, Universidad de Guadalajara, Guadalajara 44350, Jalisco, Mexico; jsergio.zepeda@academicos.udg.mx; 6 Doctorado en Ciencias de la Nutrición Traslacional, Departamento de Alimentación y Nutrición, Centro Universitario de Ciencias de la Salud, Universidad de Guadalajara, Guadalajara 44340, Jalisco, Mexico; lucrecia.carrera@academicos.udg.mx; 7 Departamento de Ingeniería Química, Centro Universitario de Ciencias Exactas e Ingenierías, Universidad de Guadalajara, Guadalajara 44430, Jalisco, Mexico; mariaa.herrera@academicos.udg.mx; 8 Bioterio, Centro de Investigación Biomédica de Occidente (CIBO), Instituto Mexicano del Seguro Social, Guadalajara 44160, Jalisco, Mexico; gerardorx@hotmail.com

**Keywords:** puerarin, obesity, metabolic dysfunction, adipose tissue inflammation

## Abstract

Obesity is a multifactorial condition characterized by adipose tissue dysfunction, insulin resistance, and low-grade systemic inflammation, contributing to metabolic disturbances. The search for natural compounds with protective actions against obesity and its complications has attracted increasing attention. Puerarin, an isoflavone derived from *Pueraria lobata*, has been reported to exert anti-inflammatory and metabolic regulatory properties. This study aimed to evaluate the effects of puerarin, a natural isoflavone, on metabolic and inflammatory alterations in a mouse model of diet-induced obesity. Male C57BL/6 mice were fed a high-fat diet (HFD) and treated orally with puerarin (50 mg/kg) for 14 weeks. The administration of puerarin resulted in a 17% reduction in weight gain, improved glucose tolerance by 6.2%, and decreased insulin resistance by 11% compared to the HFD group. Histological analysis revealed a marked reduction in hepatic steatosis and adipocyte hypertrophy. Additionally, puerarin lowered the concentration of proinflammatory cytokines, including IL-6, TNF-α, IL-1β, IL-17A, and IFN-γ, while increasing IL-10 levels. These findings suggest that puerarin may provide protective effects on glucose metabolism, liver steatosis, and adipose tissue inflammation in obesity, highlighting its possible potential as an immunometabolic modulator.

## 1. Introduction

Obesity is a global public health challenge and one of the most prevalent non-communicable diseases, with projections indicating an increase in disability, mortality, and years of healthy life lost in the coming decade [1]. It is defined by excessive fat accumulation resulting from a prolonged imbalance between energy intake and expenditure [2].

This condition is linked to ectopic lipid deposition, especially under high-fat diets, when adipose tissue exceeds its storage capacity [3]. Adipocytes respond to excess fatty acids by enlarging (hypertrophy) or increasing in number (hyperplasia), storing triglycerides via lipogenesis and mobilizing them through lipolysis during fasting or physical activity [4,5]. In individuals with obesity, sustained caloric excess promotes abnormal white adipose tissue expansion, impairing lipid handling and leading to ectopic fat accumulation in organs such as the liver and muscle. This contributes to insulin resistance, systemic inflammation, and metabolic dysfunction [6].

White adipose tissue plays a critical role in the development of chronic low-grade inflammation. When adipocytes (fat cells) enlarge, the resulting local hypoxia—caused by cell expansion—creates a proinflammatory environment [7]. This tissue is not only the primary fat reservoir but also the largest endocrine organ in the body, capable of secreting various adipokines and cytokines that have systemic effects. These molecules are involved in regulating inflammation, its resolution, and adaptive angiogenesis.

In the context of obesity, adipose tissue develops an inflammatory and dysfunctional phenotype. This condition is characterized by enlarged adipocytes, infiltration by immune cells, and an increased secretion of proinflammatory cytokines both locally and systemically. These changes impair the function of adipose tissue itself as well as the functions of distant organs [8].

Due to the complex issues associated with obesity, there is a growing interest in finding complementary therapies to help prevent and treat this condition. In this regard, naturally occurring bioactive compounds, including nutraceuticals, have gained attention for their potential to reduce the risk of non-communicable diseases such as obesity and type 2 diabetes [9].

Puerarin, also known as daidzein-8-C-glucoside (7,4′-dihydroxy-8-C-glycosyl-isoflavone), is an isoflavonoid derived from the plant *Pueraria lobata* (Willd. Ohwi) [10]. Numerous studies have documented its properties, including anti-inflammatory, antioxidant, antidiabetic, antihypertensive, anti-apoptotic, hepatoprotective, cardioprotective, and anticancer effects [11,12,13,14]. In both animal and cellular models, treatment with puerarin has been shown to reduce body weight and improve glucose and lipid metabolism, highlighting its potential as a beneficial agent for metabolic disorders [11,15].

Thus, the aim of this study was to evaluate whether puerarin supplementation can mitigate weight gain, improve glucose and insulin tolerance, reduce adipose tissue dysfunction, and modulate systemic inflammation in a murine model of high-fat diet-induced obesity.

## 2. Results

In a mouse model of obesity induced by a high-fat diet, we evaluated the effects of puerarin on key metabolic parameters and inflammatory biomarkers. This approach allowed us to investigate the molecule’s potential as a comprehensive modulator in obesity.

### 2.1. Effect of Puerarin on Body Weight

During the 14 weeks of the study, HFD animals demonstrated a progressive and significant increase in body weight compared to STD group, highlighting the sustained obesogenic effect of the diet.

When comparing total body weight gain between groups, we observed statistically significant differences. The HFD group (9.4 g ± 3 g) experienced the greatest weight gain compared to the STD group (3.24 ± 1.2 g) (*p* < 0.0001), confirming the successful establishment of the obesity model. Additionally, the administration of puerarin to animals in the STD+PUE group (3.61 ± 1 g) did not lead to a significant increase in body weight compared to the STD group. This indicates that the treatment did not affect metabolism under basal conditions.

**Figure 1:**
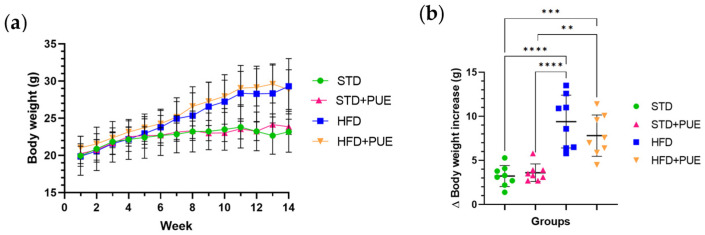
Effect of puerarin on body weight per week. (**a**) Overall body weight per week and (**b**) body weight gain. Data are shown as mean ± standard deviation, analyzed by one-way ANOVA and Tukey’s post hoc test. ** *p* < 0.01, *** *p* < 0.001, **** *p* < 0.0001, (*n* = 8 for each group).

In contrast, the HFD+PUE group (7.81 ± 2.35 g) showed a lower weight gain than the HFD group; however, it did significantly increase body weight gain compared to the STD+PUE group (*p* < 0.01) (Figure 1b).

### 2.2. The Impact of Puerarin on Glucose Metabolism

**Figure 2:**
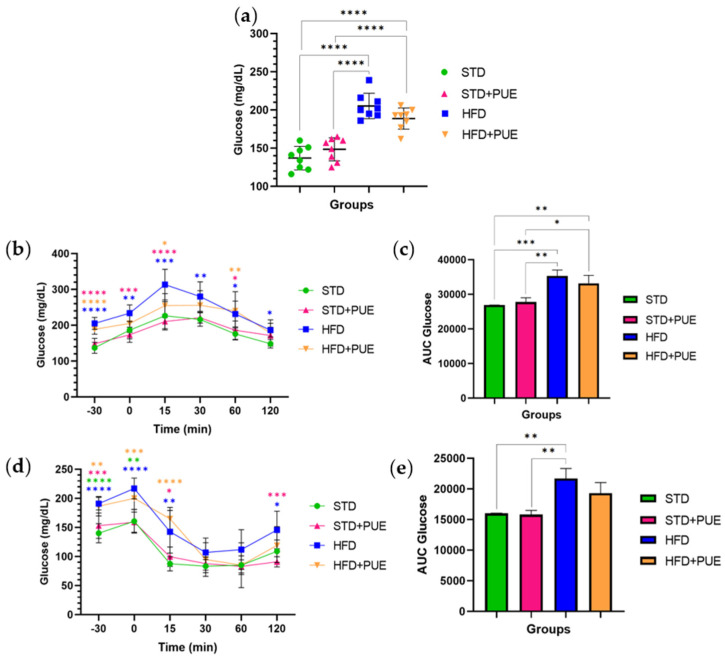
Effect of puerarin on glucose metabolism. (**a**) Fasting glucose, (**b**) Oral glucose tolerance curve, (**c**) AUC glucose tolerance curve, (**d**) Insulin tolerance curve, (**e**) AUC insulin tolerance curve. Data are shown as mean ± standard deviation, analyzed by one-way ANOVA and Tukey’s post hoc test. * *p* < 0.05, ** *p* < 0.01, *** *p* < 0.001, **** *p* < 0.0001, (*n* = 8 for each group). Asterisks indicate statistically significant differences between groups. Color-coded symbols represent each experimental group: STD (green), STD+PUE (fuchsia), HFD (blue), and HFD+PUE (orange).

Analysis of fasting glucose levels revealed significant differences among the experimental groups. The HFD group (205.25 ± 16.7 mg/dL) showed marked hyperglycemia compared to both the STD group (137 ± 15.4 mg/dL) (*p* < 0.0001) and the STD+PUE group (148.5 ± 15.1 mg/dL) (*p* < 0.0001). In contrast, animals in the HFD+PUE group (188.75 ± 13.9 mg/dL) exhibited a reduction in blood glucose levels compared to the HFD group; however, their levels did not reach the normoglycemic levels observed in the STD+PUE group (*p* < 0.0001) (Figure 2a).

The results of the oral glucose tolerance test (OGTT) showed significant differences between the experimental groups in their ability to metabolize glucose. The HFD group exhibited the highest glucose levels at all points following glucose administration, indicating impaired glycemic control. The administration of puerarin in the HFD+PUE group resulted in a partial improvement in the glycemic response, as shown by a decrease in serum glucose concentrations during the glucose tolerance test. Notably, a statistically significant reduction in glucose levels was observed at the 15 min mark when compared to the untreated HFD group (*p* < 0.05) (Figure 2b).

Furthermore, the area under the curve (AUC) analysis showed significant differences between the groups: HFD vs. STD (*p* < 0.001), HFD+PUE vs. STD (*p* < 0.01), STD+PUE vs. HFD (*p* < 0.001), and HFD+PUE vs. STD+PUE (*p* < 0.05) (Figure 2c). The HFD group displayed a higher area under the curve (AUC) value of 35,364 ± 1641. In contrast, the HFD+PUE group exhibited a reduced AUC of 33,189 ± 2302, indicating a decrease of up to 6% in the AUC during the OGTT. Meanwhile, the STD and STD+PUE groups had AUC values of 26,867 ± 970.7 and 27,779 ± 1227, respectively.

In the insulin tolerance test (ITT), animals in the STD group showed a significant decrease in glucose levels after insulin administration, indicating a normal physiological response to the hormone. In contrast, the HFD animals exhibited a reduced ability to lower blood glucose levels, with elevated levels persisting throughout the test. This outcome reflects a state of insulin resistance. The STD+PUE group exhibited a progressive decrease in glucose levels following insulin administration, indicating adequate insulin sensitivity. Conversely, the HFD+PUE group exhibited a particular behavior: at 15 min, it presented a transient peak in glucose, followed by a more pronounced decrease as the test progressed. This trend was particularly noticeable at 60 and 120 min, when their levels approached those of the control group. These results suggest that puerarin administration may be associated with a more efficient response to insulin under conditions of a high-calorie diet.

Furthermore, the AUC analysis for the ITT revealed significant differences between the HFD and STD groups (*p* < 0.01), as well as between the HFD and STD+PUE groups (*p* < 0.01) (Figure 2e). The HFD group exhibited a higher AUC value of 21,692 ± 1648. In contrast, the HFD+PUE group demonstrated a reduction in AUC, with a value of 19,312 ± 1748, indicating a decrease in the AUC of ITT by approximately 11%. Meanwhile, the STD and STD+PUE groups recorded AUC values of 16,016 ± 927.8 and 15,809 ± 685.7, respectively, indicating preserved insulin sensitivity. These findings provide further evidence of insulin resistance induced by a hypercaloric diet and suggest that this condition may be partially reversed with puerarin treatment.

### 2.3. Effect of Puerarin on the Liver

**Figure 3:**
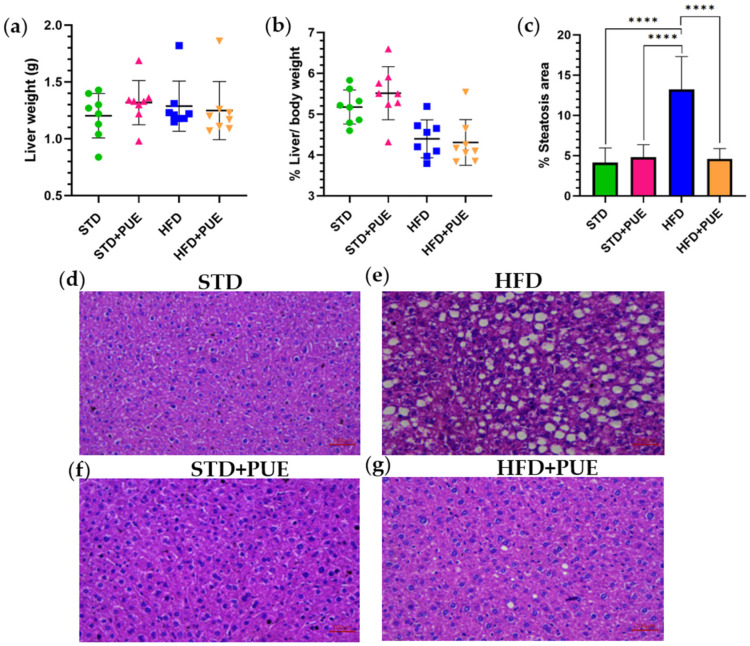
The effect of puerarin on liver morphology was assessed through several measures: (**a**) absolute liver weight, (**b**) percentage of liver weight relative to body weight, and (**c**) percentage of hepatic steatosis area. The data are presented as mean ± standard deviation and were analyzed using one-way ANOVA followed by Tukey’s post hoc test. Significance levels are indicated as follows: **** *p* < 0.0001. Representative photomicrographs of liver sections stained with H&E (20×), are displayed: (**d**) STD group; (**e**) HFD group; (**f**) STD+PUE group; and (**g**) HFD+PUE group, (*n* = 8 for each group).

During the animal sacrifice, we recorded the absolute weight of the liver tissue (Figure 3a). A slight decrease in liver weight was observed in the STD and HFD+PUE groups when compared to the HFD group; however, these differences were not statistically significant. Additionally, when we calculated the relative liver weight to body weight ratio (% liver/body weight), we found an increase in the STD and STD+PUE groups, while the HFD and HFD+PUE groups displayed relatively lower values, no significant differences were observed (Figure 3b).

Histological analysis showed a significant increase in the area of steatosis in the HFD group (13.25 ± 4.08%) compared to the STD group (4.14 ± 1.83%) (*p* < 0.0001), indicating the accumulation of lipids in the liver tissue associated with the consumption of a hypercaloric diet (Figure 3c).

In contrast, the groups treated with puerarin (STD+PUE 4.82 ± 1.36%) and HFD+PUE 4.73 ± 1.49%) exhibited a significant reduction above 35.6% of hepatic steatosis compared to the HFD group (*p* < 0.0001), with values comparable to those of the STD group. This suggests that puerarin may exert a hepatoprotective effect, likely related to its ability to modulate lipid metabolism and reduce oxidative stress.

H&E-stained histological sections reveal morphological differences among the groups. The liver parenchyma in the STD group exhibits a normal structure, characterized by uniform hepatocytes and the absence of lipid vacuolization (Figure 3d). In contrast, the HFD group exhibits a notable accumulation of lipid vacuoles in the hepatocytes, which is characteristic of macrovesicular steatosis. This validates the occurrence of fatty liver disease in animals consuming a high-fat diet (Figure 3e). The liver structure of the STD+PUE group resembles that of the STD group and shows no signs of fatty degeneration or structural alterations (Figure 3f). In the HFD+PUE group, hepatocytes show a significant decrease in the quantity and size of lipid vacuoles compared to the HFD group. These results imply that puerarin may have hepatoprotective effects against diet-induced hepatic steatosis (Figure 3g).

### 2.4. Effect of Puerarin on Adipose Tissue

**Figure 4:**
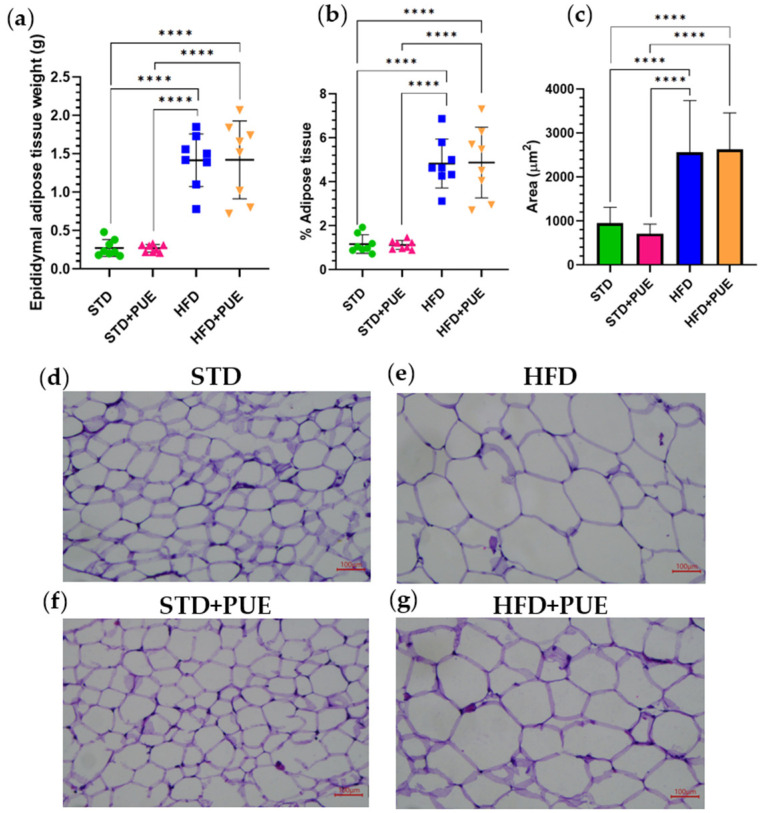
Effect of puerarin on epididymal adipose tissue morphology. (**a**) Absolute weight of adipose tissue, (**b**) Relative weight of adipose tissue, (**c**) mean adipocyte area. Data are shown as mean ± standard deviation, analyzed by one-way ANOVA and Tukey’s post hoc test. **** *p* < 0.0001, Representative photomicrographs of epididymal adipose tissue sections stained with H&E (20×), (**d**) STD group, (**e**) HFD group, (**f**) STD+PUE group, (**g**) HFD+PUE group, (*n* = 8 for each group).

Absolute and relative (% adipose tissue) weight, as well as morphometric analysis, revealed significant differences in epididymal adipocyte area between the experimental groups. HFD animals had significantly larger adipocytes than the STD group (*p* < 0.0001), confirming the development of adipocyte hypertrophy, a characteristic of diet-induced obesity; however, significant differences were observed among the following study groups: STD+PUE vs. HFD (*p* < 0.0001), STD+PUE vs. HFD+PUE (*p* < 0.0001) y HFD+PUE vs. STD (*p* < 0.0001) (Figure 4a,b).

Conversely, the administration of puerarin in the STD+PUE group (707.27 ± 222.22 µm^2^) revealed a substantial decrease in comparison to the HFD group (2563.05 ± 1176.33 µm^2^) (*p* < 0.0001) and analogous values to the STD group (947.42 ± 364.25 µm^2^). This finding indicates that puerarin does not promote adipose hypertrophy under normocaloric conditions. In contrast, the animals in the HFD+PUE group (2629.26 ± 827.97 µm^2^) did not demonstrate alterations in the adipocyte area when compared to the animals in the HFD group. However, the cells were unable to return to the levels of normalcy observed in the STD and STD+PUE groups (Figure 4c).

Photomicrographs of epididymal adipose tissue sections stained with H&E show clear differences in adipocyte morphology between the groups.

STD: adipocytes present a uniform and small size, with well-defined polygonal structures, typical of metabolically healthy adipose tissue (Figure 4d).

HFD: marked adipocyte hypertrophy is observed, with larger cells, indicating adipose tissue expansion due to excessive lipid accumulation (Figure 4e).

STD+PUE: the adipocytes maintain a similar size to the STD group, suggesting that puerarin does not alter the morphology of these cells under normocaloric conditions (Figure 4f).

HFD+PUE: adipocytes visually show a size reduction compared to the HFD group, indicating a partially protective effect of puerarin on diet-induced adipocyte hypertrophy (Figure 4g).

### 2.5. Anti-Inflammatory Effect of Puerarin

Since we were unable to obtain absolute concentrations for all analytes within the range of the standard curve, cytokine levels are reported in relative fluorescence units (RFU) as a semi-quantitative measure. These values were normalized against the control group (STD) and expressed as relative change (fold change). This method allowed us to identify trends in the inflammatory response induced by diet and influenced by treatment. However, due to these analytical limitations and the lack of absolute concentrations (pg/mL), the findings should be considered exploratory, which limits their direct biological interpretation and comparison with other studies.

**Figure 5:**
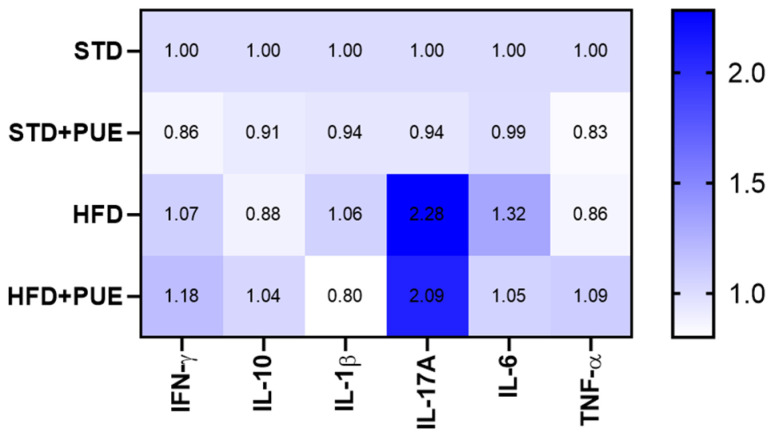
Effect of puerarin on serum cytokine profile. Heat map depicting the relative levels of proinflammatory (IFN-γ, IL-1β, IL-17A, IL-6, TNF-α) and anti-inflammatory (IL-10) cytokines in the different experimental groups, (*n* = 3 for each group).

The results of the study demonstrated that the HFD group exhibited an increase in the RFU of IFN-γ (1.07), IL-1β (1.06), IL-17A (2.28-fold higher), and IL-6 (1.32), in contrast to the STD group. While the results indicated that the HFD+PUE did not fully restore the levels of proinflammatory cytokines to their initial state, it did demonstrate an enhancement in the expression of IL-10 in comparison with the results observed in the HFD group. The STD+PUE group showed a slight but consistent decrease in all evaluated cytokines compared to the STD group, although the differences were not statistically significant. This pattern suggests that puerarin does not induce a proinflammatory response under physiological conditions (Figure 5).

Although six cytokines were evaluated, it was IL-17A that showed significant correlations with parameters relevant to the development of obesity and metabolic dysfunction.

**Figure 6:**
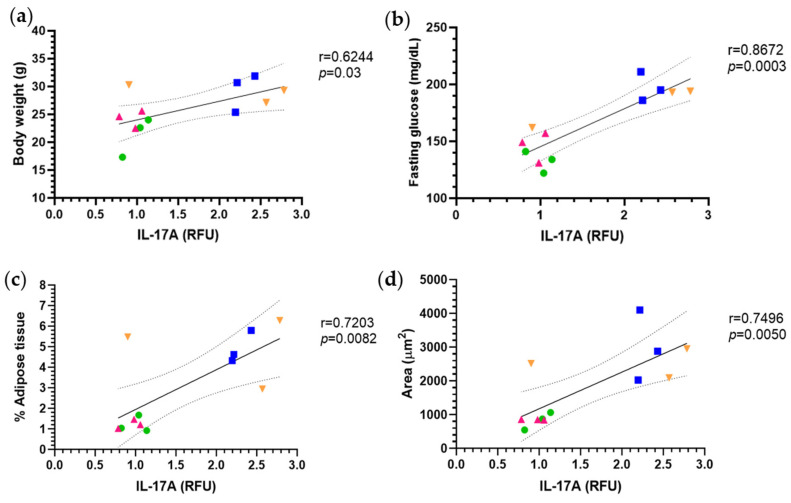
The text discusses the correlations between IL-17A levels and various metabolic parameters. Linear correlation analyses were conducted to examine the relationship between relative IL-17A levels (RFU) and the following factors: (**a**) body weight, (**b**) fasting glucose, (**c**) adipose tissue percentage, and (**d**) adipocyte area. Each data point represents an individual animal from one of the following groups: STD (green), HFD (blue), STD+PUE (pink), and HFD+PUE (orange). The results indicate a significant positive association between IL-17A levels and these metabolic parameters, suggesting that this cytokine plays an important role in immunometabolic dysfunction related to obesity, (*n* = 3 for each group).

A positive correlation was observed between IL-17A expression and body weight (r = 0.6244, *p* = 0.03), suggesting that an increase in this proinflammatory cytokine is associated with a higher degree of obesity (Figure 6a).

Likewise, a strong positive correlation was found between IL-17A and fasting glucose (r = 0.8672, *p* = 0.0003), indicating that elevated concentrations of this cytokine may contribute to the development of hyperglycemia or insulin resistance. This association reinforces the link between chronic low-grade inflammation and alterations in glucose metabolism (Figure 6b).

On the other hand, a significant positive correlation was also identified between IL-17A and the percentage of adipose tissue (r = 0.7203, *p* = 0.0082). This relationship may reflect a link between adipose tissue expansion and increased cytokine production, potentially influenced by immune cell infiltration into metabolically compromised fat depots. (Figure 6c).

Finally, a significant positive correlation was observed between IL-17A levels and adipocyte area (r = 0.7496, *p* = 0.0050). This suggests that increased expression of this cytokine in adipose tissue may be associated with greater adipocyte hypertrophy, particularly under hypercaloric dietary conditions (Figure 6d).

**Figure 7:**
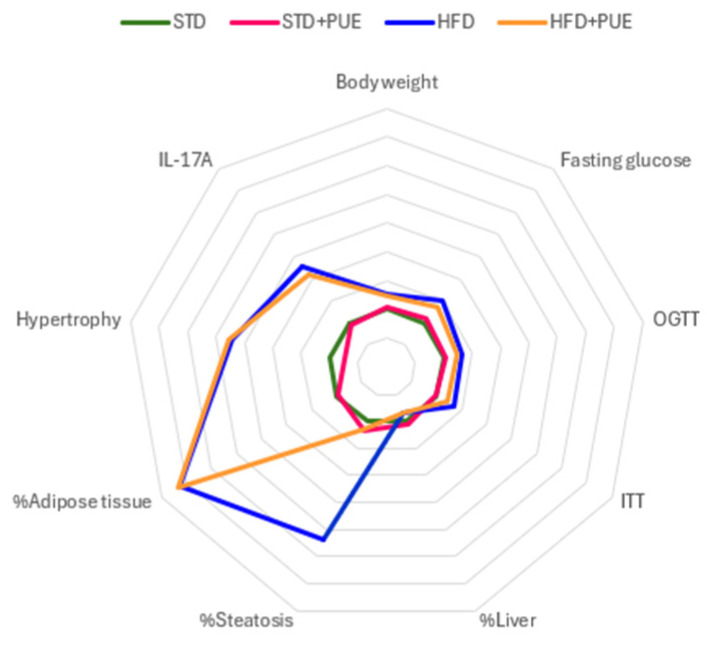
Integrative scheme of metabolic, inflammatory, and histological parameters. The radar plot shows relative values of each parameter for the STD (green), STD+PUE (fuchsia), HFD (blue), and HFD+PUE (orange) groups.

The integrative analysis of metabolic, inflammatory, and histological parameters is presented in the form of a radar graph (Figure 7), showing the relative differences between the experimental groups. The HFD group exhibited a profile characterized by pronounced increases in body weight, fasting glucose, area under the curve of the OGTT test, adipocyte hypertrophy, percentage of epididymal adipose tissue, area of hepatic steatosis, and IL-17A levels, reflecting a state of generalized metabolic dysfunction induced by HFD.

In contrast, the HFD+PUE group showed a notable reduction in the area of steatosis, although it did not manage to completely normalize the values to the level of the STD group. On the other hand, the STD and STD+PUE groups shared similar metabolic profiles, without relevant alterations in the parameters evaluated, indicating that puerarin does not exert adverse effects on homeostasis under normocaloric conditions.

This integrated analysis highlights the partial protective effect of puerarin against obesogenic diet-induced alterations, suggesting a positive impact on inflammation, lipid metabolism, and liver function in the context of obesity.

## 3. Discussion

### 3.1. Effect of Puerarin on Body Weight and Glucose Metabolism

Obesity is recognized as the consequence of a chronic energy imbalance in which caloric intake consistently exceeds the organism’s energy expenditure, favoring the excessive accumulation of adipose tissue [2]. During the progression of this pathology, white adipose tissue undergoes excessive expansion mainly mediated by adipocyte hypertrophy, because of prolonged consumption of hypercaloric diets rich in fat. This uncontrolled growth is accompanied by phenomena such as local hypoxia, programmed cell death of adipocytes, endoplasmic reticulum stress, and altered mitochondrial function. These cellular perturbations generate an inflammatory microenvironment characterized by sustained secretion of proinflammatory adipokines, a phenomenon known as chronic low-grade inflammation or meta-inflammation, which plays a central role in the development of insulin resistance, hyperglycemia, and various metabolic comorbidities, including hepatic steatosis and type 2 diabetes mellitus [8,16].

In the present study, puerarin administration to HFD-fed animals evidenced a trend toward body weight reduction. This finding differs from that reported by Noh et al., 2022 [17], who, when administering a dose of 200 mg/kg puerarin in a murine model of diet-induced obesity, observed no effect on body weight. However, on the other hand, the results are consistent with that described by Yang et al., 2016 [18], where administration of 150 mg/kg puerarin in C57BL/6 mice subjected to an HFD resulted in a significant reduction in body weight. Discrepancies between studies could be attributed to variations in the doses used, duration of treatments, and differences in the animal models used.

These results suggest that puerarin may help mitigate, to some extent, the disturbances in glucose metabolism triggered by a high-fat diet. Animals in the HFD group exhibited fasting hyperglycemia, diminished capacity for glucose metabolism, and impaired insulin responsiveness. These findings are indicative of a state of insufficiency of insulin resistance and metabolic dysfunction, which is the main characteristic of obesity. Conversely, puerarin-treated animals (HFD+PUE) demonstrated a decrease in fasting glucose levels, along with reduced areas under the curve (AUC) in glucose and insulin tolerance tests. This result points to a possible improvement in insulin sensitivity and better regulation of blood glucose levels.

These results align with recent research highlighting the hypoglycemic properties of puerarin, which encompass the reduction in hepatic glucose output, modulation of glucose transport, and stimulation of essential signaling pathways like PI3K-Akt and AMPK [19,20]. Furthermore, research suggests that puerarin may possess antioxidant properties and enhance mitochondrial function, which could contribute to its capacity to regulate glucose levels [19]. Research by Yang et al. (2016) [18] indicates that this molecule is capable of safeguarding pancreatic β-cells. The protective effects of the molecule in question are multifaceted, involving improved GLP-1 receptor signaling, increased insulin secretion, and maintained pancreatic cell mass.

### 3.2. Puerarin and the Modulation of Hepatic Lipid Metabolism

In relation to the relative liver weight to body weight, it was observed that the STD and STD+PUE groups presented values of approximately 4.6%, while the HFD and HFD+PUE groups showed proportions of around 3.6%. These values are within the physiological range reported for rodents (3–5%) [21]. It should be noted that the lower percentage observed in the HFD groups could be explained by the substantial increase in body weight, which reduces the relative proportion of the liver despite the lipid accumulation in this organ.

Although the hypercaloric diet animals did not exceed the 5% liver fat threshold associated with NAFLD [22], histological analysis by H&E staining revealed the presence of lipid vacuoles characteristic of macrovesicular steatosis. The HFD+PUE group revealed less hepatic steatosis and structural disruption, consistent with a partial protective effect of puerarin on liver tissue. These findings are consistent with that reported by Jung et al., 2017 [23], who observed that administration of 100 mg/kg puerarin decreased hepatic lipid accumulation. This effect could be associated with the ability of puerarin to modulate the activation of NF-κB and JNK pathways, which are induced by increased adipocyte-supplied free fatty acids and are involved in insulin resistance and hepatic lipogenesis [17]. Likewise, S. Wang et al., 2019 [15] reported that puerarin decreases hepatic fat accumulation via negative regulation of PARP-1 in NAFLD models.

Regarding epididymal adipose tissue, notable alterations in absolute and relative weight were detected between the groups that received an HFD and the controls, thus corroborating the consolidation of the obesity model. The ratio of body weight to epididymal adipose tissue weight was higher in the HFD groups (~3.6%) compared to the STD groups (~1.1%), evidencing the alteration of white adipose tissue linked to obesity, insulin resistance, and metabolic pathologies [24]. The application of puerarin in animals with a hypercaloric diet slightly reduced these levels.

Concerning adipocyte area, although no statistically significant difference was evident between the HFD and HFD+PUE groups, a higher prevalence of smaller adipocytes was observed in the treated animals, which could suggest a possible stimulation of adipogenesis. These results agree with those reported by Jung et al., 2017 [23], who documented a significant reduction in adipocyte area after puerarin administration. Furthermore, Li et al., 2025 [25] indicated that low doses of puerarin decrease the expression of stearoyl-CoA desaturase 1 (Scd1), a key enzyme in the desaturation of saturated fatty acids derived from de novo lipogenesis. Scd1 regulates processes such as energy homeostasis, autophagy, and inflammation, and its aberrant activation favors the progression of obesity, NAFLD, DM2, and other metabolic pathologies by modulating pathways such as AMPK/ACC and SIRT1/PGC1α [26].

In general, puerarin exerts beneficial effects on energy metabolism in obesity, favoring β-oxidation of fatty acids and glycemic homeostasis. Previous studies showed that it increases GLUT4 expression and translocation [27,28] and positively regulates AMPK and CPT-1b, enhancing lipid oxidation in skeletal muscle [29]. In addition, it reduces the expression of PARP-1, a protein involved in inflammation and lipid accumulation in the liver [15].

### 3.3. Immunomodulatory Effects and Cytokine Regulation

On the other hand, the expansion of adipose tissue in obesity generates an inflammatory microenvironment characterized by an increased infiltration of immune cells. This phenomenon has been widely described as a key factor in metabolic dysfunction [16]. Under obese conditions, adipose tissue contains a high proportion of immune cells, including macrophages, lymphocytes, and neutrophils, which secrete proinflammatory cytokines and regulate immunological homeostasis [30].

Elevated levels of IL-6, IL-1β, TNF-α, and IFN-γ have been reported in obese subjects, associated with systemic inflammation and insulin resistance [31]. Likewise, an increase in IL-17A, a cytokine that induces the expression of TNF-α, IL-6, and IL-1β [32] and promotes neutrophil infiltration in target adipose tissue, insulin resistance, and the development of NAFLD [33], has been observed in murine models.

In a complementary manner, Pearson correlation analyses were performed between the levels of inflammatory cytokines and various metabolic parameters. Particularly noteworthy was IL-17A, a proinflammatory cytokine related to the Th17 cell response, which showed a significant positive correlation with body weight, fasting glucose levels, relative epididymal adipose tissue weight, and adipocyte area, suggesting its direct involvement in obesity-induced metabolic dysfunction. These findings are consistent with recent studies that have shown that IL-17A can exacerbate inflammation and contribute to the development of non-alcoholic hepatic steatosis [32,33]. These data reinforce the relevance of IL-17A not only as a mediator of chronic inflammation, but also as a potential biomarker of metabolic alterations in obese states.

In our study, puerarin administration led to a reduction in the relative fluorescence intensity of proinflammatory cytokines IL-1β, IL-17A, and IL-6, along with an increase in the anti-inflammatory cytokine IL-10, compared to the HFD group. These changes suggest a modulatory effect on inflammatory status in the context of diet-induced obesity. These effects suggest an anti-inflammatory activity mediated by inhibition of the JNK and IKKβ/NF-κB pathways [34]. Also noteworthy is the positive correlation between IL-17A and metabolic parameters such as body weight, fasting glucose, relative adipose tissue weight, and adipocyte area, which reinforces the hypothesis that IL-17A could be considered a biomarker that plays an important role in the development of metabolic diseases.

To our knowledge, this is among the first studies to explore a quantitative association between IL-17A expression and histometabolic parameters in a high-fat diet murine model following puerarin treatment. Although research exploring the immunometabolic effects of puerarin remains limited, our findings suggest that the modulation of IL-17A may represent a key mechanism underlying its protective role. Given that IL-17A has been associated with chronic inflammation, insulin resistance, and adipose tissue dysfunction—central features in the pathophysiology of obesity—its regulation by puerarin supports the compound’s potential as an immunometabolic modulator. Taken together, our results indicate that puerarin may counteract both metabolic and inflammatory disturbances induced by a high-fat diet. Its observed ability to improve insulin sensitivity, reduce hepatic steatosis, and modulate proinflammatory cytokines reinforces its promise for future therapeutic strategies targeting obesity-related comorbidities, though further studies are still needed to confirm these effects.

#### Limitations and Future Perspectives

This study provides significant evidence regarding the effects of puerarin in a murine model of obesity induced by a high-fat diet. However, several methodological considerations should be acknowledged to guide future research.

First, the experiments were performed in male C57BL/6 mice only, which limits the generalizability of the findings, since obesity and its complications often show sex-dependent differences. Including different genetic backgrounds in future studies will help to strengthen the translational value of the results. Second, while a single dose of puerarin (50 mg/kg) was administered over 14 weeks with positive outcomes, it would be worthwhile to explore various dose-dependent therapeutic regimens over different durations. This approach could optimize efficacy and establish a more comprehensive pharmacodynamic and safety profile. Third, although key proinflammatory cytokines were assessed, additional biochemical and molecular parameters commonly linked to metabolic syndrome, such as lipid profile, liver function enzymes, insulin secretion, and oxidative stress markers, were not evaluated. Their inclusion would allow a more robust characterization of puerarin’s effects. Moreover, although previous studies suggest involvement of signaling pathways such as AMPK, PI3K-Akt, and NF-κB, these were not directly examined here. Future work incorporating transcriptomic, proteomic, or targeted molecular analyses will be crucial to confirm the intracellular mechanisms modulated by puerarin, particularly in adipose and liver tissues. The underlying molecular mechanisms were not thoroughly investigated. Future research could incorporate transcriptomic or proteomic methodologies to elucidate better the intracellular signaling pathways modulated by puerarin, particularly in adipose and liver tissues.

We propose to investigate the impact of puerarin on lipid metabolism, its interaction with incretins, and its potential role in maintaining the integrity of the intestinal barrier. Additionally, we will explore how puerarin influences microbiota and modulates the gut-microbiota-brain axis, which could reveal new systemic effects of this compound. This research aims to provide evidence regarding the systemic effects of puerarin. These inquiries will contribute to establishing its potential as an adjuvant agent in comprehensive therapeutic strategies for addressing obesity and its associated comorbidities. Furthermore, they may support the rational design of future translational or clinical studies.

## 4. Materials and Methods

Experimental, comparative, and longitudinal study using a murine model of obesity with mice of the C57BL/6 strain, developed at the Centro Universitario de Ciencias Exactas e Ingenierías (CUCEI), Universidad de Guadalajara.

### 4.1. Puerarin

The puerarin (PUE) (98% purity) used in this protocol was acquired from Biosynth (Staad, Switzerland) Cat # MP08125.

### 4.2. Diet

A standard diet was administered, which provided 4.07 kcal/g, consisting of 18.3% protein, 59.6% carbohydrate, and 22.1% fat (Basal Diet 5755, Labdiet, St. Louis, MO, USA). In contrast, the diet high in saturated fat contained 5.1 kcal/g, with 18.1% protein, 20.3% carbohydrate, and 61.6% fat (DIO Rodent Purified Diet with 60% energy from fat-Blue 58Y1, Labdiet, St. Louis, MO, USA).

### 4.3. Animals

Male mice of the C57BL/6 strain were used in this study. Although this could be considered a gender bias, several studies have documented that females of this strain are more resistant to the development of obesity induced by a high-calorie diet, mainly attributed to protective hormonal and metabolic factors [35]. For this reason, male mice were chosen to create a more sensitive and consistent model for studying obesity and its comorbidities.

Thirty-two male C57BL/6 strain mice, weighing between 20 and 25 g, were obtained from the Centro de Investigación Biomédica de Occidente (CIBO) in Guadalajara, Mexico. The animals were kept at a regulated temperature of 25 °C with a 12 h light–dark cycle, having access to food and water freely, except during tests that necessitated fasting. The current research project has been approved by the Comité Institucional para el Cuidado y Uso de Animales de Laboratorio (CICUAL), Centro Universitario de Ciencias Exactas e Ingenierías (CUCEI) of Universidad de Guadalajara, with registration number CUCEI/CINV/CICUAL-03/2022 (approved on 7 September 2022). All animal procedures were performed by the guidelines stipulated in NOM-062-ZOO-1999 [36], which delineates the technical specifications for the production, care, and use of laboratory animals.

### 4.4. Experimental Design

**Figure 8:**
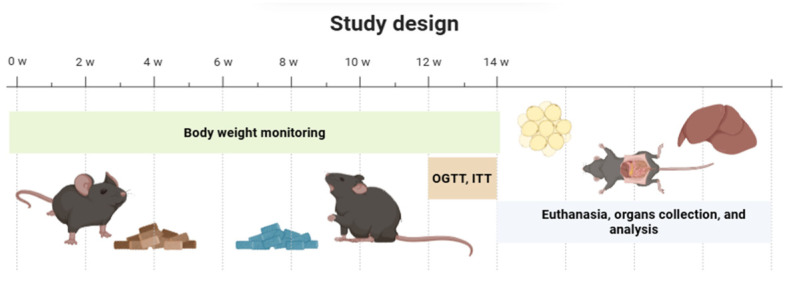
Experimental design.

After 3 weeks of acclimatization, they were divided into two types of standard (STD) and hypercaloric diet (HFD) and randomly classified into four groups: STD (*n* = 8), STD+PUE (*n* = 8), HFD (*n* = 8), and HFD+PUE (*n* = 8). A dose of 50 mg/kg PUE was administered orally via orogastric cannula for 14 weeks, which was dissolved in physiological saline solution (PSS) with 4% Tween 80 (Sigma Aldrich, St. Louis, MO, USA). However, to avoid biases related to handling conditions, the untreated STD and HFD groups received the same solution used as a vehicle to dissolve puerarin orally. This administration was performed using an orogastric cannula, thus ensuring that all experimental groups were subjected to the same handling procedure and associated stress (Figure 8).

It is important to note that the dose selected for this study was based on the results of a pilot trial, which evaluated the biological effects of puerarin at concentrations of 50 mg/kg and 100 mg/kg. This preliminary analysis indicated greater efficacy at the 50 mg/kg dose, particularly regarding body weight, fasting glucose levels, and responses to the oral glucose tolerance test (OGTT).

While previous studies have utilized higher doses, a significant methodological difference is found in the delivery systems used; many rely on specialized formulations or more invasive routes [37,38,39,40]. In our study, we opted for direct oral administration to minimize animal handling. However, this decision required us to address the low solubility of the puerarin molecule. To improve solubility, we used a micelle-based formulation prepared with physiological saline solution and 4% Tween 80. This formulation demonstrated enhanced solubility, making it the chosen vehicle for administering the treatment.

### 4.5. Increased Body Weight and Increased Deposits of Adipose and Liver Tissue

Weekly monitoring of the body weight of each mouse was performed, using a digital scale with a deviation of ±0.01 g. At the end of the protocol, the body weight gain was calculated; the weights of week 1 and week 14 were considered. During the sacrifices, the adipose tissue and liver deposits were weighed, using a Grantaria balance with a ±0.01 g deviation.

### 4.6. Fasting Glucose

The measurement of fasting glucose is useful to monitor the metabolic state of an individual, since it indicates the capacity of the cells to absorb and use energy substrates, as well as their capacity to maintain their homeostasis in the circulation. For this measurement, the animals were fasted for 4 h, then blood was obtained by tail puncture, and glucose levels were measured with the Accu-chek Instant equipment (Roche, Basel, Switzerland).

### 4.7. Oral Glucose Tolerance Test and Insulin Tolerance Test

The oral glucose tolerance test (OGTT) was performed at the end of the 14 weeks of experimentation. Mice were fasted for 3.5 h, and glucose was measured (−30′) to rule out biases in the molecule concentration due to stress generated by handling the mice during the measurement. After 4 h of fasting, time zero (0′) was considered, glucose was measured again, in addition to subsequently administering 150 μL of a glucose solution (2 g glucose/kg VO of mouse) and, serum glucose measurements were taken at −30′, 0′, 15′, 30′, 60′ and 120′ min.

On the other hand, for the insulin tolerance test (ITT), the same procedure described above was followed; however, 0.75 IU/kg of insulin was injected intraperitoneally instead of the glucose solution, after 4 h of fasting.

### 4.8. Adipose Tissue and Liver Morphology

Adipose tissue and liver were obtained during sacrifice and preserved in a 4% paraformaldehyde solution, then sent to a certified pathologist for further analysis, who conducted hematoxylin and eosin staining. A total of eight mice were used to perform the histological sections. Once the slides were stained, the images were captured using an optical microscope. To ensure a representative sample, five fields were analyzed per slide, resulting in a total of 160 photomicrographs (40 for each group), which were later analyzed automatically through ImageJ 1.54g software.

### 4.9. Cytokines

Serum cytokine levels were quantified using the MULTI-PLEX technique with the Bio-Plex Pro™ Mouse Cytokine Th17 Panel A 6-Plex Kit^®^ (Cat. No. M6000007NY, Bio-Rad Laboratories, Hercules, CA, USA), following the manufacturer’s instructions. Before analysis, samples were diluted 1:4 by mixing 40 µL of serum with 120 µL of assay diluent. Each sample was analyzed once; no technical replicates were included.

### 4.10. Statistical Analysis

The sample size (*n*) was determined utilizing G*Power 3.1.9.7 software [41,42], featuring a power of 80%, a significance level of 0.05, and based on the information provided by Noh et al., 2022 [17], which yielded an effect size of 7.57 for the average difference in hepatic steatosis between the NC and PUE groups, which required a sample size of 16 animals. Similarly, a second sample size was determined for the adipocyte size variable, with an effect size of 1.03 for the difference between the NC and PUE groups, resulting in an *n* = 2. In this context and considering the 3R principle (Replacement, Reduction, and Refinement in animal research), a decision was made to select a moderate sample size (*n* = 8) for each group in the experiment. The Shapiro–Wilk normality test was utilized to assess the distribution of quantitative variables. The one-way ANOVA test and Tukey’s post hoc test for multiple comparisons were utilized for group comparisons. When the normality assumptions were not satisfied, the nonparametric Kruskal–Wallis test was employed, with Dunn’s test applied for post hoc analysis.

For correlation analyses, Pearson’s or Spearman’s correlation coefficient was used according to the normal distribution of the variables (body weight, fasting glucose, relative adipose tissue weight, adipocyte area, and IL-17A). The aim was to explore relationships between variables of metabolic dysfunction and inflammatory profile.

Statistical analyses were performed using GraphPad Prism^®^ version 6 software (GraphPad Software Inc., San Diego, CA, USA). A value of *p* < 0.05 was considered statistically significant.

## 5. Conclusions

In summary, our findings show that puerarin has a comprehensive protective effect against metabolic, inflammatory, and histological changes caused by a high-fat diet. The use of puerarin decreased hyperglycemia during fasting, optimized glucose tolerance and insulin sensitivity, decreased lipid accumulation in the liver, and attenuated adipose tissue dysfunction, manifested by a reduction in relative tissue weight, and release of proinflammatory cytokines such as IL-1β, IL-6, and IL-17A.

Conversely, the decrease in IL-17A, which is strongly associated with negative metabolic factors, suggests that this cytokine may serve as a biomarker for immunometabolic alterations. Therefore, puerarin is a promising candidate for medicinal strategies that aim to prevent and treat diabesity and its related comorbidities. Nevertheless, additional research is necessary to more thoroughly comprehend the molecular mechanisms underlying these effects, encompassing the function of puerarin in regulating intestinal microbiota composition and function. Additionally, well-designed clinical trials are needed to confirm their efficacy and safety in human subjects.

Puerarin mitigates obesity-induced metabolic dysfunction in high-fat diet-fed mice by reducing adipocyte hypertrophy, lipid accumulation, and proinflammatory cytokine production. These effects may involve modulation of the adipose tissue immune microenvironment, highlighting puerarin’s potential as an immunometabolic modulator in obesity.

## Data Availability

The data presented in this study are available on request from the corresponding author.

## Abbreviations

The following abbreviations are used in this manuscript:

AUC	Area Under Curve
HFD	High-Fat Diet
ITT	Insulin Tolerance Test
OGTT	Oral Glucose Tolerance Test
RFU	Relative Fluorescence Units
STD	Standard Diet
